# Study protocol of a parent-focused child feeding and dietary intake intervention: the feeding healthy food to kids randomised controlled trial

**DOI:** 10.1186/1471-2458-12-564

**Published:** 2012-07-28

**Authors:** Kerith Duncanson, Tracy Burrows, Clare Collins

**Affiliations:** 1 Nutrition and Dietetics, School of Health Sciences, Faculty of Health, The University of Newcastle, Callaghan, NSW, 2308, Australia; 2 Hunter New England Local Health District, Forster, NSW, 2428, Australia; 3 Priotity Research Center in Physical Activity and Nutrition, The University of Newcastle, Callaghan, NSW, 2308, Australia

**Keywords:** Dietary intake, Child/ren, Feeding, Parent, Nutrition education, Resources, Randomised controlled trial

## Abstract

**Background:**

Poor childhood nutrition is a more pervasive and insidious risk factor for lifestyle-related chronic disease than childhood obesity. Parents find it difficult to address the reported barriers to optimal child feeding, and to improve child dietary patterns. To impact at the population level, nutrition interventions need to be easy to disseminate, have a broad reach and appeal to parents while overcoming the barriers parents face when trying to improve child feeding behaviours. The Feeding Healthy Food to Kids (FHFK) Randomised Control Trial (RCT) examines the impact of providing low cost, self-directed nutrition and parenting resources to rural parents, on child dietary intake and parent–child feeding practices.

**Methods/Design:**

Up to 150 parents of two-to-five year old children will be recruited in five rural Australian towns. Eligible, consenting parents will be randomly allocated to intervention or 12-month wait-list control groups. Intervention group parents will receive an interactive nutrition CD and parenting DVD, and be provided with instructions for optimal resource utilisation. Intervention and control group participants will also receive a generic nutrition and physical activity brochure and a physical activity resource to blind participants to group allocation. Primary outcome measures are dietary intake of vegetables (serves/day), fruit and energy dense nutrient poor foods (serves/day and %Energy). Secondary outcome measures are total energy (kCal), other food groups (serves/day and %Energy), key nutrients (mg/day), child feeding domains and parenting style domains.

Analysis of dietary outcome measures, child feeding and parenting domains will be conducted on an intention-to-treat basis and compared at baseline, three and 12 months using the random effects model, using STATA software. Details of the methodological aspects of recruitment, inclusion criteria, randomisation and statistical analysis are described.

**Discussion:**

This paper will add to existing research examining child feeding practices and dietary intake of young children, by specifically focusing on the efficacy of an RCT that has the potential to be implemented at a population level. The correlation of the RCT outcomes with parents’ perceptions about child feeding practices and children’s dietary intake of their children in a subsequent qualitative study will further contribute to this emerging area of research.

**Trial registration:**

Australian Clinical Trials Registration Number: ACTRN12609000356268

## Background

Poor childhood nutrition is a more pervasive and insidious risk factors for chronic disease than childhood obesity [1]. Eating habits established in childhood track through to adulthood, so a childhood diet that is dominated by energy-dense, nutrient-poor foods, low in fruit and vegetables is likely to persist into adulthood [2-6]. The two most common dietary inadequacies reported in analysis of young children’s dietary intake within Australia [7-10] and internationally [8,11,12] are inadequate total consumption (and variety) of vegetables [5,8,10-13] and excess consumption of energy-dense, nutrient-poor foods and beverages [8,10,12,14]. Both are risk factors for increased risk of chronic conditions, including specific cancers, type II diabetes and cardiovascular disease (CVD) [6,15,16].

As the ‘gate-keepers’ of the child’s eating environments [3,17-19], parents are important agents of dietary behaviour development. This is particularly true for pre-school children aged two to six years, as a large proportion of their food is consumed within the home environment [5,20]. Engagement of parents is therefore critical in any early childhood nutrition intervention [21,22].

Parents consistently report child nutrition to be amongst their highest priorities and responsibilities [21,22]. However, a disparity exists between parental desire for a balanced diet for their child/ren and actual child dietary intake [23]. Factors contributing to poor dietary intake amongst children include poor maternal nutrition knowledge [9,24] and role modelling of energy-dense, nutrient-poor food consumption [9,25,26]. Energy-dense, nutrient-poor foods can easily be misrepresented in food advertising as being core foods [27,28]. Additionally, parents tend not to make a connection between children’s dietary intake and longer term health consequences in the absence of obvious risk factors, such as childhood obesity [29]. Parents also underestimate the weight status of their own children, and are often unaware of what body weight and height proportion constitutes overweight and obesity [30].

Birkett [31] has reported that parent concern for disease prevention, home food availability and parental attitudes, beliefs and practices related to child feeding all impact significantly on a child intake. Maternal self-efficacy has also recently been reported to positively impact on child eating behaviours while authoritative parenting style has consistently been associated with optimal child feeding and eating patterns [4,32,33].

Dietitians and public health experts invest considerable time and effort in developing nutrition resources and education programs for parents of young children [34,35]. Although nutrition interventions should be grounded in health behaviour theory [36], this is rarely the case. They are often ‘stand alone’ or single-strategy interventions, and their efficacy is rarely evaluated in the community or population setting [37,38].

Effective nutrition education resources and programs need to have a broad reach, and appeal to parents. It is vital that these strategies promote enablers, and overcome the barriers to parent engagement in health behaviour change, particularly those related to child feeding and nutrition [37,39]. Web-based and self-directed resources could replace face-to-face education to improve reach and engagement and their development has been recommended [31,40]. Two studies from the US Supplemental Nutrition Program for Women, Infants and Children (WIC) program provide strong evidence for a self-directed educational approach [31,41].

In rural areas of Australia there is reduced availability of and access to paediatric nutrition health services [38,42-44] Therefore, a self-directed nutrition program is an attractive option for parents and health care providers who reside in rural areas [44]. Increasingly, rural people are becoming technology literate and have improved access to computers, electronic home entertainment and high speed internet connections [40]. Interactive nutrition and parenting resources have the capacity to be provided across whole populations.

### Study aim

The aim of this study is to describe the study protocol of a randomised controlled trial that is designed to determine the efficacy of providing self-directed nutrition education resources to rural parents.

## Methods/Design

### Ethics approval

Approval for the study was obtained from Hunter New England (HNE) Human Research Ethics Committee and the University of Newcastle Human Ethics Research Committee in 2009.

HNEHREC Reference No: 08/12/17/4.02 NSW HREC Reference No: HREC/08/HNE/403.

HNE SSA Reference No: SSA/08/HNE/UoN; H-2009-0106.

The trial was registered with the Australian Clinical Trials Registration in 2009, trial number ACTRN12609000356268.

Written informed consent was obtained from all participants prior to their enrolment in the FHFK study.

### Design - randomized control trial

A prospective randomized control trial (RCT) to measure the impact of providing parents of pre-school aged children with self-directed nutrition and parenting resources on selected dietary and child feeding factors variables, following intention-to-treat principles, with secondary per-protocol analysis.

### Selection criteria

The criteria for inclusion in the RCT are shown in Table 1.

**Table 1:** Inclusion and exclusion criteria for recruitment into the feeding healthy food to kids randomized controlled trial

**Inclusion Criteria**	**Exclusion Criteria**
Parent aged 18 years or over (mother, father or primary carer)	Parent under 18 years old
	Child aged under two years or over six years
Eldest child in family who is aged between two and five years (inclusive)	Child commenced primary school
	Chronic health condition that significantly impacts on child’s dietary intake
Parents from designated study localities or surrounding areas.	
	Additional study children from same family

#### Recruitment

Participants will be recruited primarily from Children’s Services (Long Day Care Centres, Pre-schools, Family Day Care, in-home care, Playgroups) and by Early Childhood health professionals who have direct contact with parents of children in target age group, and work in study locations. The primary recruitment strategy will be reinforced by strategic dissemination of flyers and newspaper advertisements in order to maximise the effect of successive approximation, whereby potential participants are exposed to the study more than once, to increase likelihood of participation.

#### Randomization

**Figure 1:**
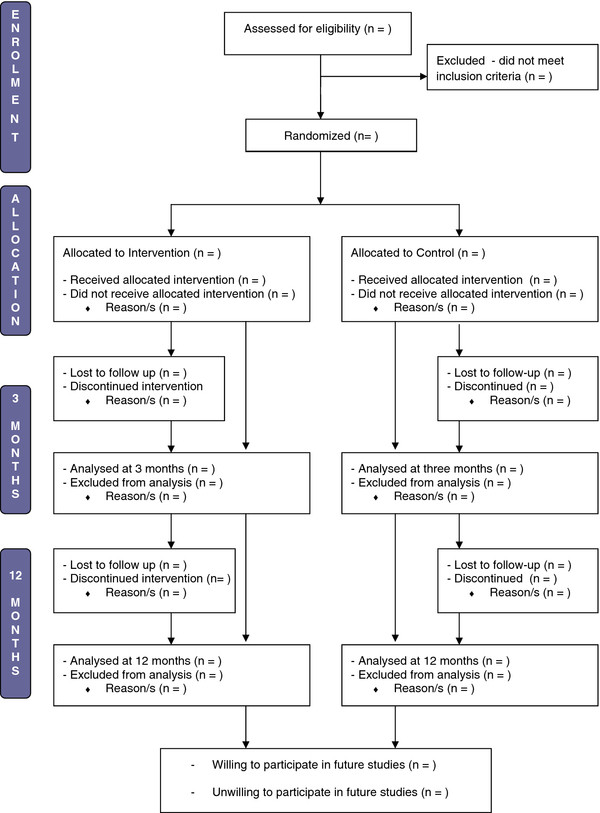
Flow of participants through the feeding healthy food to kids randomized controlled trial.

Returned baseline survey envelopes will be numbered sequentially from one to 150 before being opened. A computerised random number generator will be used to generate 75 random numbers between 1 and 149, which constitute the ‘intervention’ numbers, with the remaining numbers being the ‘control’ numbers. Allocation of participants to the control or intervention group will be conducted by one member of the research team (KD), who is blinded to participant identity. Participants will be blinded to group allocation throughout the trial. Randomisation numbers will only be used to assign participants to their group, before switching to the use of unique research codes based on location, research phase and consent form numbers. The flow of participants through the trial is outlined in Figure 1.

### Sample size

Vegetable and energy-dense, nutrient-poor food (and beverage) consumption are the primary outcomes of FHFK. Vegetable consumption was used to determine sample size because evidence suggests this to be the most challenging dietary factor to change [9,11,38]. Based on 80% power to detect a significant difference between the intervention and control groups of 37.5 grams (0.5 serves) of vegetables (*P* = 0.05, two-sided), a sample size of 100 participants is needed at 12 months. Allowing for a drop-out rate of 30%, up to 150 participants will be recruited.

### Theoretical framework for FHFK study design

Changing the dietary intake of children requires change to the feeding practices of parents. The Theory of Planned Behaviour (TPB), originally developed to predict and explain human social behaviour, is being used in FHFK to serve as a framework for a behaviour change interventions. In the context of FHFK, the key components of the TPB are proposed to predict the child feeding practices or behaviours of parents. Table 2 summarises how TPB has been applied to change parental behaviour in order to increase child intake of vegetables within the FHFK trial.

**Table 2:** Application of the theory of planned behaviour to feeding healthy food to kids randomized controlled trial

**Theoretical component**	**FHFK application**	**FHFK component (measurement tool)**
Personal beliefs + evaluation = attitude towards behaviour	· Personal beliefs about feeding children	· RC parent interviews (LSAC, CFQ)
		· TR modules 1, 2, 3, 4 (ATES) and RC child feeding section
	· Personal evaluation of children’s dietary intake	
		· RC parent interviews (LSAC, CFQ
	· Attitude towards child feeding	
Normative beliefs + motivation to comply with norms = Subjective norm	· Normative beliefs about child feeding	· RC celebrity parent interviews
		· RC child section - feedback, fussy eating, encouragement, consistency (CFQ, LSAC)
	· Normative beliefs about children’s dietary intake	
	· Motivation to comply with dietary guidelines	· TR modules 1, 2, 3, 4, 7, 8, 9, 10 (ATES, CFQ)
Perceived behavioural control and behavioural intention	· Perceived control over child feeding and intention to change child feeding or the dietary intake of children	· TR Module 7 (Fussy eating) RC child section for parental efficacy (LSAC) and child feeding (CFQ)

TR = Tummy Rumbles, RC = Raising Children DVD, LSAC = Longitudinal Study of Australian Children,

CFQ = Child Feeding Questionnaire, ATES = Australian Toddler Eating Survey Food Frequency Questionnaire.

### Resource selection

The resource selection process for use in the FHFK study involved three sequential steps.

1) An initial review of literature to identify key features of successful nutrition interventions (see Table 3).

2) Mapping of available Australian nutrition and parenting resources against key success factors and capacity for application of resources as a low intensity population level intervention.

3) Mapping of outcome measures for FHFK RCT against resource modules/sections; vegetable consumption: reducing energy-dense, nutrient-poor foods: parenting skills: and child feeding practices (see Table 2).

**Table 3:** Enablers, barriers and resources for parent engagement in health behaviour change

**Enablers of parent engagement in behavioural change**	**Barriers to engaging parents in behavioural change**
· Collaborative, whole of agency approach	· Delayed response to identification of issues
· Recognition of mutual expertise	· Feelings of isolation or victimisation
· Belief that parents are trying their best	· Fear of being labelled a ‘bad parent’
· Target various stages of readiness to change	· Fear of failure
· Importance of ‘engaging’ parents	· Parents unaware of consequences of behaviours
· Multi component strategies, multiple referral methods	· Parents ambivalent to change own behaviours
· Low level interventions for simple behavioural change	· Inadequate time allowance between exposure and expected adoption of health behaviour change
· Encourage authoritative parenting	
· Normalise parenting support	
**Enablers of optimal child feeding and childhood nutrition**	**Perceived barriers to optimal child feeding (parent cited)**
· Role modelling healthy eating habits	· Lack of information about overcoming fussy eating Inadequate communication about nutrition from childcare Impact of food marketing Poor food availability and confusion about food labelling
· Involvement of children in food preparation	
· Availability of reputable resources in the public domain	
· Early intervention and a theoretical basis for programs	· Food used as a reward despite parent knowledge
· Universal interventions for less severe needs,	· Perceived lack of appropriate nutrition resources
· Parents receptive to/capable of behavioural change	· Need for ‘one stop shop’
· Targeting parenting skills in addition to nutrition	· Need for user friendly resources related to healthy eating
· Programs that encourage authoritative parenting styles, with or without a nutrition or child feeding focus	
· Multifaceted and community wide programs	
**Effective health education resource/strategy components**	**Ineffective health education resource/strategy components**
· Educational home visits or telephone education	· Printed materials of limited value
· ‘Parents as teachers’ model	· Didactic approach to teaching
· Resources that are socially and culturally appropriate	· Lack of consideration for adult learning principles
· Educational resources need to be reading age appropriate	· Poor training of educators to work parents in paediatrics
· Web resources to replace face-to-face education	· Resources not appropriate for target group
· Ensure ample ‘dosage’ of technology resources	
· Use of internet for rural participants	
· Optimal balance of regulation, legislation and education	

While none of the reviewed resources contained all of the nutrition and parenting elements measured in FHFK, two resources were selected for use together. The evaluation of resources by three research team members indicated that the combination of these two resources provided an optimal balance of parenting and nutrition information in a desirable format for an information technology intervention, with capacity to be applied across a population.

The Tummy Rumbles [45,46] interactive nutrition education CD is a self-directed resource that was adapted from an early childhood nutrition education program for Childcare staff and parents. The resource is divided into modules that include: the five food groups, dietary fats, fussy eaters, healthy lunchbox ideas, food budgeting and reading food labels. It has been evaluated by users as a useful and effective resource for early childhood nutrition education [46].

Raising children [22] is ‘a guide to parenting from birth to 5’, the content of which is based on the principles of the Raising Children [22] website*,* Australia’s definitive parenting resource. It contains different sections for parents of newborns, baby and child. Participants in this study were requested to view the DVD’s child section, specifically the segments on eating strategies, junk food, encouraging behaviour, minimising choking risk, play and learning.

The resources selected for the control group were provided to participants with the intention of enhancing retention of control group participants, providing placebo contact with researchers, preventing resentful demoralisation due to not receiving active intervention [47] and blinding the participants to study group allocation. “*Here’s 3 steps you should know*…” is a generic three-fold brochure that is widely available across the study locations, and promotes three key health messages; drink water, get active and eat fruit and vegetables [48]. *Active alphabet*[49] is a set of two books, one targeted at parents and one version specifically for use by children. They are colourful, informative and provide an A to Z guide to increasing physical activity with young children through development of fundamental movement skills.

### Intervention

Participants who complete baseline surveys and are randomised will be sent their respective intervention or control group resources by mail within one week of receipt of baseline surveys. They will be followed up by their preferred communication method (telephone call, text message or email) after another week to ensure that the resources had been received. Resources are accompanied instructions explaining how to access the information on the interactive resources. Minimal guidance regarding the recommended frequency of utilisation will be given, to mimic real-life dissemination of resources, and to simulate how parents might access resources within a low intensity intervention.

Participants will receive a reminder by telephone, text or email about using the resources when the three month surveys are administered. No further prompting of participants to use the resources will be provided prior to the final data collection at 12 months. At the trial conclusion participants will be sent an order form for a range of free resources for participating in the study (see Table 4). These include the Tummy Rumbles CD and Raising Children DVD (for control group participants), plus a recipe book and supermarket pocket guide. The order form also contains a section to be completed by participants if they are willing to be involved in further studies related to the Feeding Healthy Food to Kids RCT.

**Table 4:** Time line and participant requirements for FHFK study

**Phase**	**Study action**	**Intervention group parents**	**Control group parents**
**Promotion**	Distribute Flyers	Flyer received	Flyer received
**Recruitment**	Distribute PIS + consent	Completed consent form	Completed consent form
**Baseline data**	Distribute surveys	Demographics	Demographics
	Collect surveys Randomisation	ATES, CFQ	ATES, CFQ
	Baseline data analysis		
**Resources**	Distribution of resources	Raising Children	‘3 steps’ brochure
		Tummy Rumbles	Active Alphabet
		‘3 steps’ brochure	
		Active Alphabet	
**3 month data**	Distribute surveys	ATES, CFQ	ATES, CFQ
	Reminder call, text, email	Resource feedback	
	Collect surveys, data analysis		
**12 month data**	Distribute surveys	ATES, CFQ	ATES, CFQ
	Reminder call, text, email	Resource feedback	Resource feedback
	Collect surveys, data analysis		
**Resources**			Raising Children
			Tummy Rumbles

PIS = Participant Information Survey ATES = Australian Toddler Eating Survey CFQ = Child Feeding Questionnaire.

### Data collection procedures

Parents who provide written consent to participate will be mailed baseline surveys; the Australian Toddler Eating Survey (ATES) Food Frequency Questionnaire (FFQ), Child Feeding Questionnaire, the parenting questions from the Longitudinal Study of Australian Children (LSAC) questionnaire, demographic data questionnaire, instructions for the completion and return of surveys, and a stamped return envelope. Participants will be informed in the instructions for completion that the surveys will take a total of 30 to 45 minutes to complete. A period of six weeks will be allocated for return of surveys. If the surveys are not returned after four weeks, participants will receive a reminder via their preferred contact method (phone call, text message or email) to encourage them to return the surveys. Returned survey envelopes will be numbered for blinded randomisation prior to opening envelopes. The researchers will then check incoming surveys for completeness, and contacted participants for clarification or completion of missing questions.

Three and twelve month data collection involves repeated administration of all questionnaires administered at baseline, except for the demographic data questionnaire being excluded and a resource utilisation questionnaire being included (see Table 4).

### Quality control

Quality control procedures will be employed to optimise the quality of the study and maximise validity and reliability of the program delivery and outcome assessments. These include:

**Assessor blinding:** Assessors of the main outcome measures are blinded to participant group allocation. Food frequency questionnaires will be checked for completeness and sent directly to the University of Newcastle by data collection personnel. Child feeding and parenting questionnaires will be analysed by an individual other than the assessor.

**Written documentation:** All written documentation, including assessment protocols and letters sent to participants, are standardised and subject to institutional ethics committee approval.

**Training:** Data collection personnel will be trained prior to data collection. Standardised procedures for data cleaning will be documented and provided to data collection personnel. Where possible, the same assessors will be used for all assessments.

**CONSORT guidelines:** The study protocol followed the CONSORT guidelines for Randomised Controlled Trials.

### Measurement instruments and outcome measures

#### Demographic data

Data used to establish the demographic profile of the study population will be collected using a demographic data questionnaire at baseline.

#### Demographic outcome measures

Categorical responses will be reported for the following outcome measures:

Parent: age group (five year increments), education level and Aboriginal Torres Strait Islander status

Study child: age group (six month increments), type of child care, Aboriginal Torres Strait Islander and health status

### Child dietary intake

Dietary intake will be assessed using the Australian Toddler Eating Survey (ATES), a 120-item semi-quantitative food-frequency questionnaire (FFQ). The ATES is currently being tested for reliability and relative validity [50] and demonstrates acceptable accuracy for ranking nutrient intakes in Australian toddlers 2 to 4 years. Additional validation studies are currently underway.

The ATES tool is used by parents to record their child’s frequency of consumption of a comprehensive, defined list of foods over the previous six month period. Each question contains between four and eight categorical responses. Parents will complete the ATES at baseline, 3 months and twelve months. The ATES surveys are computer analysed and each response is converted from the frequency of serves into an estimated gram weight, by auto calculation of serves, using the median intake per serve from the Australian National Nutrition Survey.

Nutrient intakes from the FFQ will be computed from the Australian AusNut 1999 database (All Foods) Revision 17 and AusFoods (Brands) Revision 5 (Australian Government Publishing Service, Canberra) to generate individual mean daily macro-and micro-nutrient intakes, and percentage Energy (%E) contributions of foods or foods groups.

Questions relating to specific food items from the FFQ will be aggregated categorically based on food groups corresponding to the Australian Guide to Healthy Eating: breads and cereals, fruits, vegetables, dairy foods, meat or alternatives and “extras” (energy-dense, nutrient-poor foods). The energy-dense, nutrient-poor foods category will be broken down into sweetened drinks, sweet packaged snacks, savoury packaged snacks, baked goods, confectionery, take-out foods and fatty meats. Sub categories are consistent with previous studies [51,52].

Serves of fruits and vegetables will be calculated by summing the weight of food items in the FFQ coded as fruits or vegetables and dividing by the serve size dictated in the Australian Guide to Healthy Eating (fruits; 150 g and vegetables; 75 g). All other foods in the ATES are quantified using multiples of standard children portions from the 1995 Australian National Nutrition Survey of Children and Adolescents or Foodworks computer nutrition analysis tool.

### Dietary intake outcome measures

Primary outcome measures are: Vegetables consumption (serves/day); Fruit consumption (serves/day and %Energy (E)); Total Energy-dense, nutrient-poor foods food intake (serves/day and %E).

Secondary outcome measures are: Total Energy (kJ and kCal); Dairy intake (serves/day and %E), Bread, grains and cereals (serves/day and %E), Meat and alternatives (serves/day and %E), Micronutrients: Iron and calcium (mg/day).

### Child feeding measurement instrument

Parents will complete the self-report, 31–item Child Feeding Questionnaire (CFQ) [17] at baseline, three and 12 months to identify whether the provision of resources influenced child feeding practices. The CFQ is designed for use by parents of children aged two to 12 years. Items are measured using a five-point Likert-type scale, with each point on the scale represented by a word anchor. Parental beliefs and attitudes regarding child feeding practices are measured in seven domains; perceived responsibility (mean of three items), parent perceived weight (mean of four items), perceived child weight (mean of three items), parents' concerns about child weight (mean of three items), monitoring (mean of three items), restriction (mean of eight items), pressure to eat (mean of four items).

Scores for each question in each of seven domains are aggregated and divided by the number of questions in each respective domain. Mean scores for each participant can be compared with nationally representative data, and within the study at each time point and between groups.

### Child feeding outcome measures

Perceived responsibility

Parent perceived weight

Perceived child weight

Parents' concerns about child weight

Monitoring

Restriction

Pressure to eat

### Parenting

The Longitudinal Study of Australian Children (LSAC) [53] parenting questions will be used to evaluate parenting across a variety of domains. Six domains from the parenting section of the LSAC dataset will be analysed in the FHFK RCT. *Warmth, Inductive Reasoning, Parental Efficacy* and *Overprotection* are assessed using a 1-5 point Likert scale with responses ranging from ‘never/almost never’ (=1) to ‘always/almost always’ (=5). *Warmth* is a 6-question domain that identifies the level of affection that a parent displays towards their child. *Inductive Reasoning* is a 3-question domain that analyses a parent’s level of communication related to discipline. *Parental Efficacy* is a 4-question domain that identifies child behaviours related to parental discipline. *Overprotection* is a 3-question domain that identifies how much a parent attempts to shelter their child.

*Self-efficacy* is measured by a single question that asks parents how they feel, using a 5-point Likert scale ranging from ‘a very good parent’ (=1) to ‘not very good at being a parent” (=5). *Parental Hostility* is 5-question domain that assesses how often parents display anger related behaviour towards their child. This domain is measured on a 10-point Likert scale ranging from ‘not at all’ (=1) to ‘all the time’ (=10).

Scores for each question in each of the four selected domains are aggregated and divided by the number of questions in each respective domain. Mean scores for each participant can be compared with nationally representative data, and within the study at each time point and between groups.

### Parenting outcome measures

Inductive Reasoning

Parental Efficacy and

Self Efficacy

Overprotection

Hostility

### Statistical analysis

Demographic data, child feeding and parenting data and resource evaluation data will be manually entered into STATA by one of the researchers (KD). The ACAES surveys will be cleaned and scanned in a customised nutrition analysis program and imported into STATA format for analysis. Normality checks on all data will be conducted prior to further analysis. No imputation of missing values will be carried out for subjects; the study and subjects will be analysed in their allocated, randomisation group.

Analysis will be on an intention-to-treat basis. Differences in vegetable consumption, fruit consumption, total energy-dense, nutrient-poor foods food intake will be analysed using the random effects model, with the effects being time (baseline, three and 12 months), group (intervention or control) and group-by-time interaction. Statistical significance will be set at P < 0.05. Statistical analysis will be completed using STATA statistical software (Version 10, College Station, Texas USA). The primary intention-to-treat analysis involved all participants who are randomly assigned and complete baseline and 3 month and/or twelve month surveys, regardless of whether they reported using the resources.

Secondary per-protocol analysis will be conducted to determine the relative impact of resource utilisation. Participants will be considered to have adhered to the study protocol if they report using Tummy Rumbles for at least one hour and Raising Children for at least one hour over the course of the 12-month trial. The control group will have met the protocol if they report using Active Alphabet at least once within the 12-month trial. The same analysis protocol will be followed after removal of data for participants who did not follow the study protocol.

## Discussion

It is critical to evaluate whether nutrition education tools and strategies meet the needs of parents, and if this leads to changes in child feeding behaviours that are reflected in measureable changes in child dietary intake. Data demonstrating whether the study yields positive long-term results will provide direction for future health promotion and research and increase the capacity for the implementation of well-designed RCTs to address the lack of quality low intensity nutrition interventions targeted at parents of young children in rural areas.

The FHFK study is one of the first randomized controlled trials to determine the impact of a population level nutrition resource intervention on the dietary intake of Australian children. The FHFK RCT extends current research into early childhood nutrition by (i) examining the child feeding practices of parents in a community setting, using a theory based intervention model that could be applied at a population level (ii) having a 12-month follow-up period, thereby allowing assessment of medium term intervention effectiveness; (iii) including several important secondary outcomes (child feeding practices, parenting styles, dietary intake), and (iv) inclusion of a representative sample of rural parents of children aged two to five years, a demographic group previously under-represented in childhood nutrition studies.

The study builds on previous research into the role of parents in children’s dietary intake. Although studies in Australia and the USA have examined the impact of intensive nutrition interventions on the dietary intake of toddlers and primary school aged children, there has not been an extensive study of dietary intake of children aged between two and five years, particularly at a population level and particularly those living in rural areas. Although numerous studies [13,51,54] have identified changes in dietary intake as a result of face-to-face education, and other studies [44] have use web based programs to influence parent’s child feeding practices, little analytic attention has been paid to influencing children’s dietary intake through low intensity interventions based on distribution of low cost resources that can be delivered at a population level.

Feeding Healthy Food to Kids addresses this issue by analysing the dietary intake of two to five year old children, using a validated food frequency questionnaire that has been completed by parent proxy. As such, this study provides additional insight into the potential for a parent-focused nutrition intervention to influence the dietary intake of rural pre-school aged children. The analytic focus on the relative contribution of core food groups and energy-dense, nutrient-poor foods, and comparison of actual dietary intake with child feeding and parenting practices adds a further contribution to the body of research in the field of children’s dietary intake and child feeding.

## Competing interests

All authors declare that they have no competing interests.

## Authors’ contributions

CC, KD, TB were responsible for the design of study, the dietary and child feeding intervention and this paper. All authors were responsible for drafting the manuscript and have read and approved the final version.

## Pre-publication history

The pre-publication history for this paper can be accessed here:

http://www.biomedcentral.com/1471-2458/12/564/prepub
